# Rapamycin inhibits Erk1/2-mediated neuronal apoptosis caused by cadmium

**DOI:** 10.18632/oncotarget.4087

**Published:** 2015-05-25

**Authors:** Chong Xu, Hai Zhang, Chunxiao Liu, Yu Zhu, Xiaoxue Wang, Wei Gao, Shile Huang, Long Chen

**Affiliations:** ^1^ Jiangsu Key Laboratory for Molecular and Medical Biotechnology, Jiangsu Key Laboratory for Microbes and Functional Genomics, College of Life Sciences, Nanjing Normal University, Nanjing, PR China; ^2^ Department of Biochemistry and Molecular Biology, Louisiana State University Health Sciences Center, Shreveport, LA, USA; ^3^ Feist-Weiller Cancer Center, Louisiana State University Health Sciences Center, Shreveport, LA, USA

**Keywords:** rapamycin, neuronal apoptosis, PP2A, PTEN, Erk1/2

## Abstract

Cadmium (Cd), an environmental contaminant, causes neurodegenerative disorders. Recently we have shown that rapamycin prevents Cd-induced neuronal cell death by inhibiting mTOR signaling pathway. Here we found that rapamycin exerted its prevention against Cd-induced neuronal cell death also partially via blocking Erk1/2 pathway. Inhibiting Erk1/2 with PD98059 or silencing Erk1/2 potentiated rapamycin's inhibition of Cd-induced phosphorylation of Erk1/2 and apoptosis in neuronal cells. Both PP2A and PTEN/Akt were involved in the regulation of Erk1/2 activation and cell death triggered by Cd. Inhibition of PP2A with okadaic acid or ectopic expression of dominant negative PP2A attenuated rapamycin's inhibition of Cd-induced phospho-Erk1/2 and apoptosis, whereas over-expression of wild-type PP2A enhanced rapamycin's effects; Over-expression of wild-type PTEN or dominant negative Akt, or inhibition of Akt with Akt inhibitor X strengthened rapamycin's inhibition of Cd-induced phospho-Erk1/2 and cell death. Furthermore, expression of a rapamycin-resistant and kinase-active mTOR (mTOR-T) blocked rapamycin's inhibitory effects on Cd-induced inhibition of PP2A, down-regulation of PTEN, and activation of Akt, leading to Erk1/2 activation and cell death, whereas silencing mTOR mimicked rapamycin's effects. The results uncover that rapamycin inhibits Cd activation of Erk1/2-mediated neuronal apoptosis through intervening mTOR-PP2A/PTEN signaling network.

## INTRODUCTION

Cadmium (Cd), a toxic heavy metal with very strong accumulation in human body, has a long biological half-life (15–20 years) mainly due to its low rate of excretion from the body [1]. Clinical and epidemiological data have shown that Cd exerts toxic effects not only on the kidneys [2], liver [3, 4], lung [5] and testis [6] but also on the central nervous system (CNS) [7–9]. Prolonged exposure to Cd results in the dysfunction of CNS such as learning disabilities and hyperactivity in children, olfactory dysfunction, and neurobehavioural defects in attention, psychomotor speed and memory in workers [9–12]. Cerebral cortical and hippocampal neurons have been identified as targets of Cd toxicity and Cd-induced cell apoptosis [8, 12–14]. Overwhelming evidence has demonstrated that Cd-poisoning is a possible etiological factor in neurodegenerative diseases, such as Parkinson's disease (PD), Alzheimer's disease (AD) and amyotrophic lateral sclerosis [7, 9, 12, 15].

The mammalian target of rapamycin (mTOR), a serine/threonine (Ser/Thr) kinase, functions as two complexes (mTORC1 and mTORC2) in mammalian cells [16, 17]. mTORC1 phosphorylates ribosomal p70 S6 kinase 1 (S6K1) and eukaryotic initiation factor 4E (eIF4E) binding protein 1 (4E-BP1) [16, 17], whereas mTORC2 phosphorylates Akt on Ser473 [18]. In addition to phosphorylation on Ser473, Akt activity is also positively regulated by phosphorylation on Thr308 by phosphoinositide-dependent kinase 1 (PDK1), which requires activation of phosphatidylinositol 3′-kinase (PI3K) [16–19]. Activated Akt also positively regulates mTOR, leading to increased phosphorylation of S6K1 and 4E-BP1 [20, 21]. The PI3K-Akt-mTOR pathway is negatively regulated by PTEN (phosphatase and tensin homologue on chromosome 10), a dual specificity protein and lipid phosphatase [20–22]. Though more functions of the mTOR complexes may be identified, current knowledge indicates that mTOR plays a crucial role in the regulation of cell growth, proliferation, differentiation, survival, and motility [16, 17].

Numerous studies have demonstrated that mTOR regulates differentiation and survival in neurons, and plays an important role in synaptic plasticity, learning and memory, and food uptake in adult brain [23, 24]. mTOR activity is modified in various pathologic states of the nervous system, including brain tumors, tuberous sclerosis, cortical displasia and neurodegenerative disorders such as PD, AD, and Huntington's disease (HD) [24]. Active Akt, as a major regulator of neuronal cell survival [25], is negatively associated with dopaminergic neurodegeneration in PD [26, 27]. We have recently found that Cd induces neuronal apoptosis via down-regulation of PTEN and activation of Akt/mTOR signaling pathway [12, 20, 28, 29]. Rapamycin, a macrocyclic lactone, is a potent and specific mTORC1 inhibitor [30]. Mounting data have indicated that mTORC1 is sensitive to short rapamycin exposure [16, 17]. However, the effect of rapamycin on mTORC2-mediated Akt phosphorylation depends on the concentration and duration of rapamycin treatment [31]. Our group has demonstrated that pretreatment with rapamycin *in vitro* for 48 h prevents Cd-induced neuronal cell death by inhibiting Akt/mTOR signaling pathway [20]. Administration of rapamycin *in vivo* also potently attenuates Cd-induced activation of Akt/mTOR signaling, brain damage and neuron death in mice [12].

In mammalian cells, there exist at least three distinct groups of MAPKs, including the extracellular signal-regulated kinases ERK1/2, ERK3/4, ERK5, ERK7/8, the Jun N-terminal kinases JNK1/2/3, and the p38 MAPKs p38α/β/γ/δ [32]. Multiple studies have reported that sustained activation of Erk1/2, JNK and/or p38 MAPK contribute to Cd-induced apoptosis in various types of cells, including neuronal cells [33, 34]. Our previous studies have shown that all three MAPK members can be activated by Cd in neuronal cells, and Cd-induced neuronal apoptosis is only partially attributed to activation of Erk1/2 and JNK, but not p38 [28]. As protein phosphatases 2A (PP2A) negatively regulates Erk1/2 pathway through dephosphorylation of Erk1/2 [35], we have also found that Cd induces activation of Erk1/2 contributing to neuronal apoptosis via inhibition of PP2A activity [36]. As mentioned above, PTEN negatively regulates Akt/mTOR pathway [22, 29, 37]. We have observed that Cd can down-regulate PTEN protein expression, leading to activation of Akt/mTOR signaling in PC12 cells [20]. Interestingly, emerging evidence has suggested that PTEN may also negatively regulate Erk1/2 pathway in several malignancies [38]. In addition, PI3K/Akt may activate Erk1/2 through PKC [38]. mTOR negatively regulates PP2A, and rapamycin can activate PP2A [39]. Based on the above findings, we hypothesized that rapamycin inhibits Cd activation of Erk1/2 pathway via activating PP2A and PTEN network, thereby preventing neuronal cell apoptosis.

Here we show that rapamycin inhibits Cd-induced neuronal cell death in part by suppressing Erk1/2 pathway. Mechanistically, rapamycin blocks Cd activation of Erk1/2, not only by preventing Cd inhibition of PP2A, but also via blocking Cd down-regulation of PTEN and activation of Akt in neuronal cells in an mTOR kinase activity-dependent manner. Our findings underline a potential beneficial role of rapamycin in the prevention and/or treatment of Cd-induced neurodegenerative disorders.

## RESULTS

### Rapamycin attenuates Cd-induced neuronal apoptosis by blocking Erk1/2 pathway

**Figure 1:**
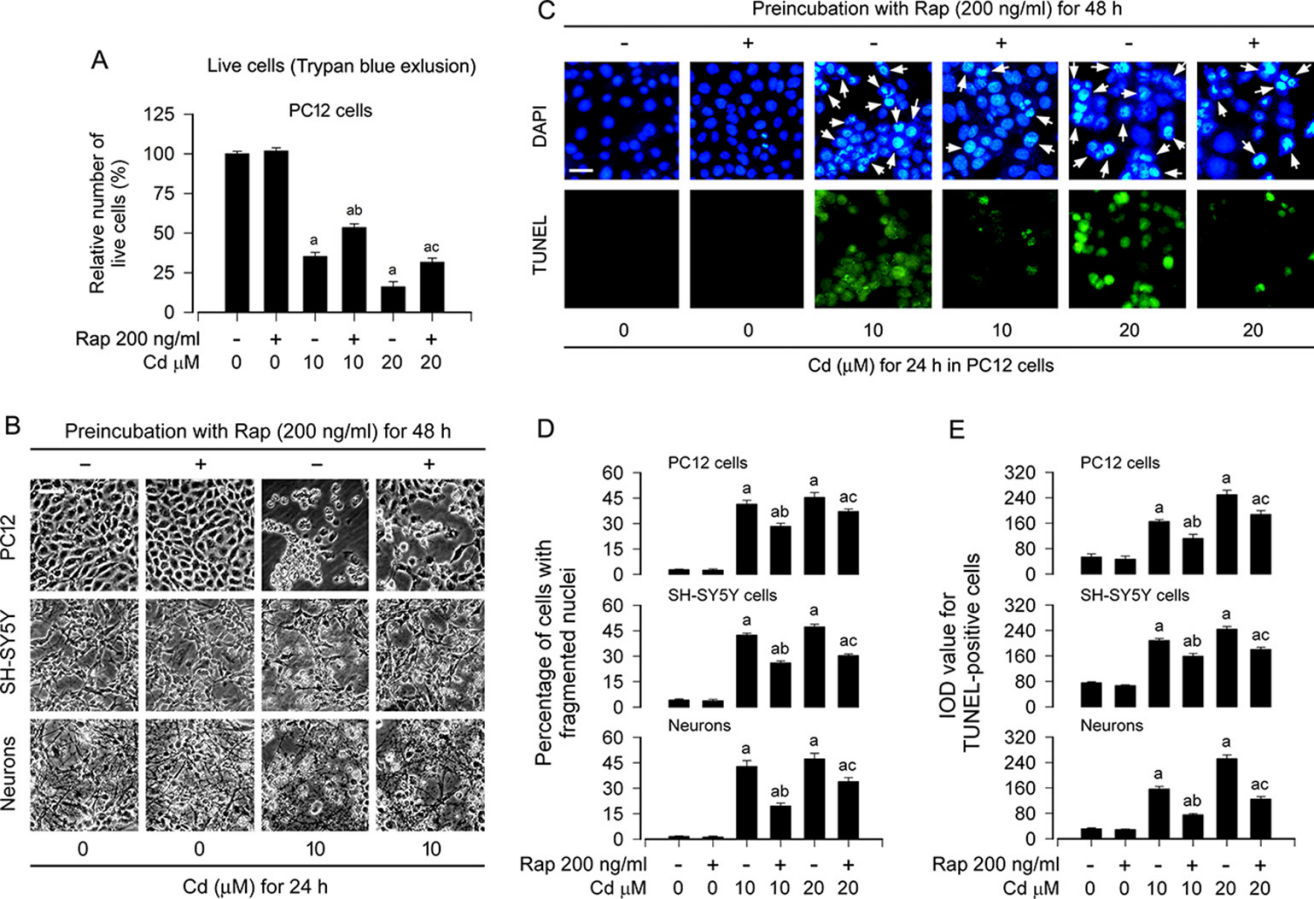
Rapamycin attenuates Cd-induced apoptotic cell death in neuronal cells PC12 cells, SH-SY5Y cells and primary neurons were pretreated with rapamycin (Rap, 200 ng/ml) for 48 h, and then exposed to Cd (10 and/or 20 μM) for 24 h. **A.** Live cells were detected by counting viable cells using trypan blue exclusion. **B.** Cell morphological changes were visualized under an Olympus inverted phase-contrast microscope (200 ×) equipped with Quick Imaging system. Scale bar: 50 μm. **C.** Apoptotic cells were evaluated by nuclear fragmentation and condensation (arrows) using DAPI staining (upper panel) and concurrently by *in situ* detection of fragmented DNA (in green) using TUNEL staining (lower panel). Scale bar: 20 μm. **D and E.** The percentages of cells with fragmented nuclei (D) and the number of TUNEL-positive cells (E) were quantified, showing that rapamycin dramatically attenuated Cd-induced apoptosis in PC12 cells, SH-SY5Y cells and primary neurons. For (A), (D), and (E), all data were expressed as means ± SE (*n* = 5). ^a^*P* < 0.05, difference with control group; ^b^*P* < 0.05, difference with 10 μM Cd group; ^c^*P* < 0.05, difference with 20 μM Cd group.

We have recently demonstrated that Cd induces neuronal apoptosis in part through activation of mTOR/MAPK signaling network [28, 36, 40], and inhibition of mTOR by rapamycin *in vitro* and *in vivo* prevents Cd-induced neurotoxicity [12, 28]. In line with the above findings, here we also observed that pretreatment with rapamycin (200 ng/ml) for 48 h attenuated the cell viability reduction and morphological change induced by 24-h exposure to Cd (10 and/or 20 μM), as detected by trypan blue exclusion in PC12 cells (Figure 1A) and morphological analysis in PC12 cells, SH-SY5Y cells and primary neurons (Figure 1B), respectively. Next, we evaluated the cells with nuclear fragmentation and condensation, a hallmark of apoptosis [41], using DAPI staining, and concurrently analyzed DNA strand breaks in the cells by TUNEL staining (Figure 1C). Imaged and quantified results showed that pretreatment with rapamycin significantly reduced the percentage of the cells with nuclear fragmentation and condensation (arrows) and the number of TUNEL-positive cells with fragmented DNA (in green) in PC12 cells, SH-SY5Y cells and primary neurons triggered by Cd exposure, compared with the control (Figure 1C–1E).

**Figure 2:**
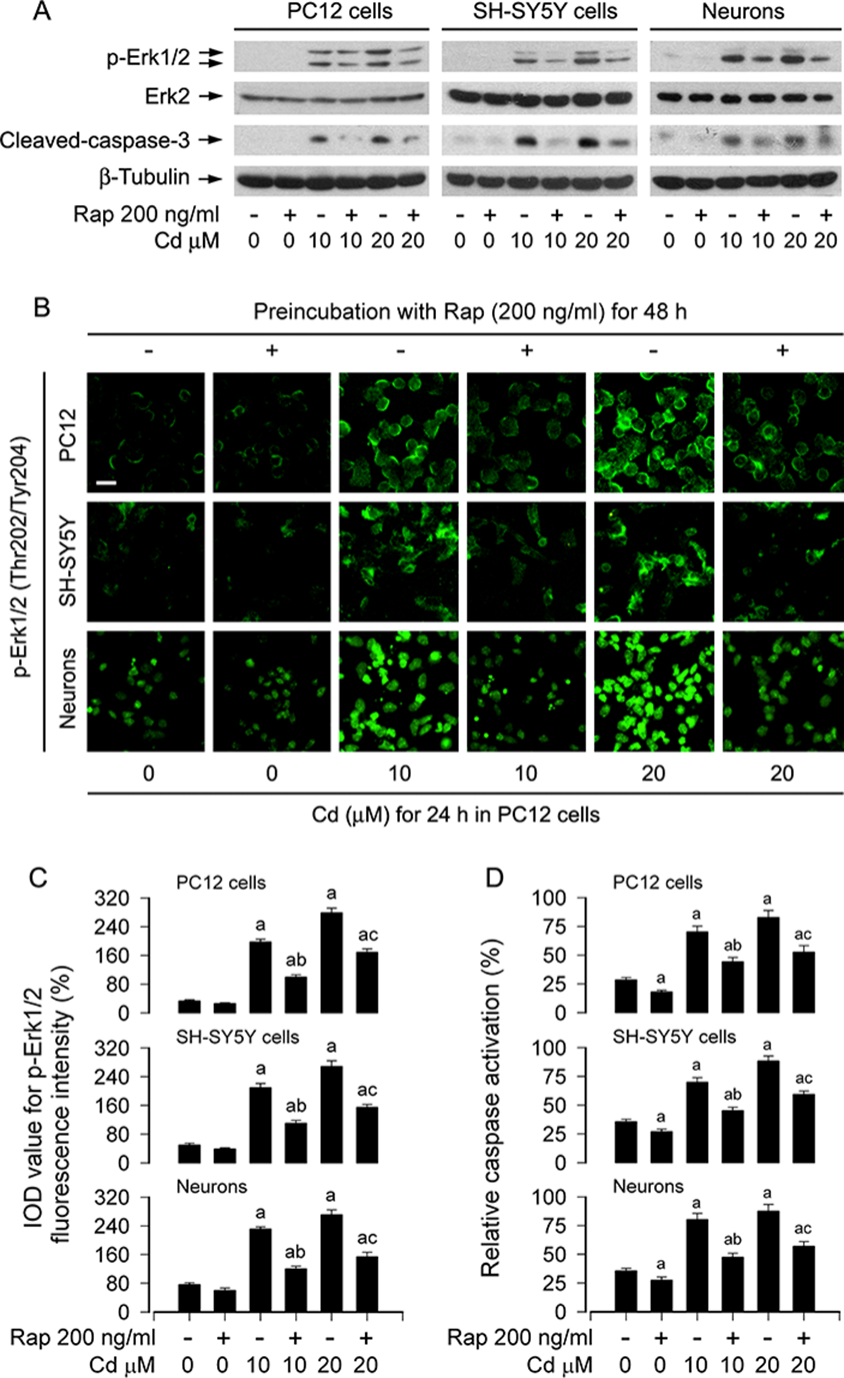
Rapamycin inhibits Cd-induced activation of Erk1/2 and caspases in neuronal cells PC12 cells, SH-SY5Y cells and primary neurons were pretreated with rapamycin (Rap, 200 ng/ml) for 48 h, and then exposed to Cd (10 and 20 μM) for 4 h (for Western blotting) or 24 h (for immunofluorescence staining and caspase-3/7 activity assay). **A.** Total cell lysates were subjected to Western blotting using indicated antibodies. **B and C.** Expression of p-Erk1/2 (Thr202/Tyr204) is imaged using immunofluorescence staining, showing that treatment of the cells with Cd for 24 h resulted in higher p-Erk1/2 expression (in green), which was obviously attenuated by rapamycin. Scale bar: 20 μm. **D.** Caspase-3/7 activity was determined using Caspase-3/7 Assay Kit, showing that rapamycin substantially blocked Cd activation of caspases 3/7 in the cells. For (A), the blots were probed for β-tubulin as a loading control. Similar results were observed in at least three independent experiments. For (C) and (D), all data were expressed as means ± SE (*n* = 5). ^a^*P* < 0.05, difference with control group; ^b^*P* < 0.05, difference with 10 μM Cd group; ^c^*P* < 0.05, difference with 20 μM Cd group.

In addition, using Western blot analysis, we also investigated the cleavage of caspase-3 in PC12 cells, SH-SY5Y cells and primary neurons. The results revealed that rapamycin potently blocked Cd-elicited robust cleavage of caspase-3 in the cells (Figure 2A). Interestingly, we also found that rapamycin obviously suppressed Cd-induced phosphorylation of Erk1/2 (Figure 2A), hinting that rapamycin may inhibit Cd-induced activation of Erk1/2 pathway, preventing Cd-induced cell death. To confirm this finding, we next conducted p-Erk1/2 immunofluorescence staining and caspase3/7 activity assay in PC12 cells, SH-SY5Y cells and primary neurons, respectively. Treatment of the cells with Cd (10 and 20 μM) for 24 h induced remarkable phosphorylation of Erk1/2 (Thr202/Tyr204) (in green), which was obviously diminished by rapamycin pretreatment (Figure 2B and 2C). Consistently, rapamycin substantially blocked Cd-induced activation of caspases 3/7 in the cells (Figure 2D).

**Figure 3:**
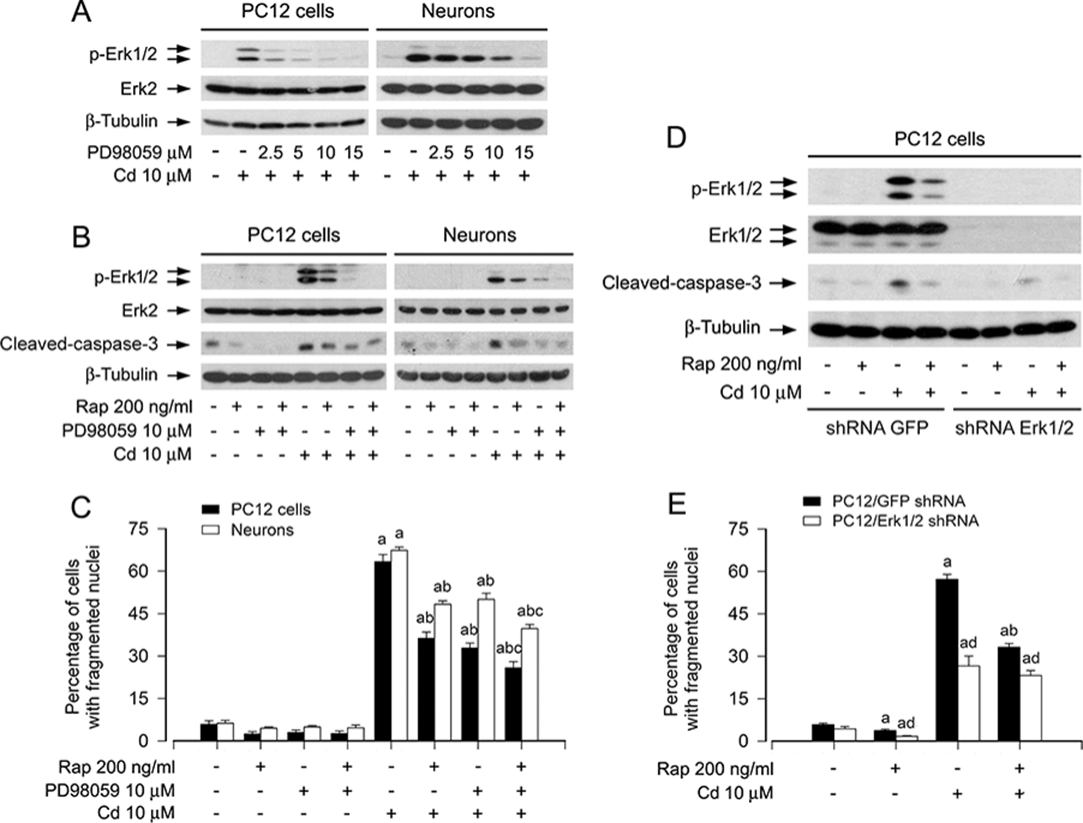
Pharmacological inhibition of Erk1/2 with PD98059 or down-regulation of Erk1/2 strengthens rapamycin's prevention of Cd-induced apoptosis in neuronal cells PC12 cells and primary neurons, or PC12 cells infected with lentiviral shRNA to Erk1/2 or GFP (as control), respectively, were pretreated with/without PD98059 (2.5–15 μM) for 1 h, pretreated with/without rapamycin (Rap, 200 ng/ml) for 48 h and then with/without PD98059 (10 μM) for 1 h, or pretreated with/without Rap for 48 h, followed by exposure to Cd (10 μM) for 4 h (for Western blotting) or 24 h (for live cell assay and cell apoptosis analysis). **A, B and D.** Total cell lysates were subjected to Western blotting using indicated antibodies. **C and E.** Apoptotic cells were evaluated by nuclear fragmentation and condensation using DAPI staining. For (A), (B), and (D), the blots were probed for β-tubulin as a loading control. Similar results were observed in at least three independent experiments. For (C) and (E), all data were expressed as means ± SE (*n* = 5). ^a^*P* < 0.05, difference with control group; ^b^*P* < 0.05, difference with 10 μM Cd group; ^c^*P* < 0.05, difference with Cd/Rap group or Cd/PD98059 group. ^d^*P* < 0.05, Erk1/2 shRNA group versus GFP shRNA group.

To validate the observation that rapamycin inhibition of Cd-induced cell apoptosis is linked to the blockage of Erk1/2 activation, PC12 cells and primary neurons were pre-incubated with/without PD98059 (a selective inhibitor of MAPK kinases 1/2 (MEK1/2), upstream of Erk1/2) alone, or in combination with rapamycin. We found that PD98059 (2–15 μM) concentration-dependently inhibited Cd-induced phosphorylation of Erk1/2 in the cells (Figure 3A). PD98059 (10 μM) or rapamycin (200 ng/ml) alone obviously suppressed the phosphorylation of Erk1/2 and the cleavage of caspase-3 in the cells induced by Cd exposure (Figure 3B). Especially, co-treatment with rapamycin/PD98059 exhibited a stronger inhibitory effect on Cd-induced phospho-Erk1/2 and cleaved-caspase-3 (Figure 3B). Consistently, the combination of rapamycin with PD98059 also exhibited more potent inhibition of Cd-triggered cell apoptosis than rapamycin or PD98059 alone (Figure 3C).

To further corroborate the role of Erk1/2 in rapamycin's inhibition of Cd-induced neuronal apoptosis, Erk1/2 was silenced by RNA interference. As detected by Western blotting (Figure 3D), lentiviral shRNA to Erk1/2, but not to GFP, down-regulated expression of Erk1/2 by ~90% in PC12 cells. Silencing Erk1/2 almost completely blocked Cd-induced phosphorylation of Erk1/2 and cleavage of caspase-3 (Figure 3D). Consistently, down-regulation of Erk1/2 conferred significant resistance to Cd-induced apoptosis in PC12 cells as well (Figure 3E). Importantly, addition of rapamycin rendered the cells more resistant to Cd treatment (Figure 3D and 3E). The results clearly indicate that rapamycin attenuates Cd-induced apoptosis, at least in part by blocking Erk1/2 pathway in neuronal cells.

### Rapamycin prevents Cd-induced activation of Erk1/2 and apoptosis in part by blocking Cd inhibition of PP2A in neuronal cells

**Figure 4:**
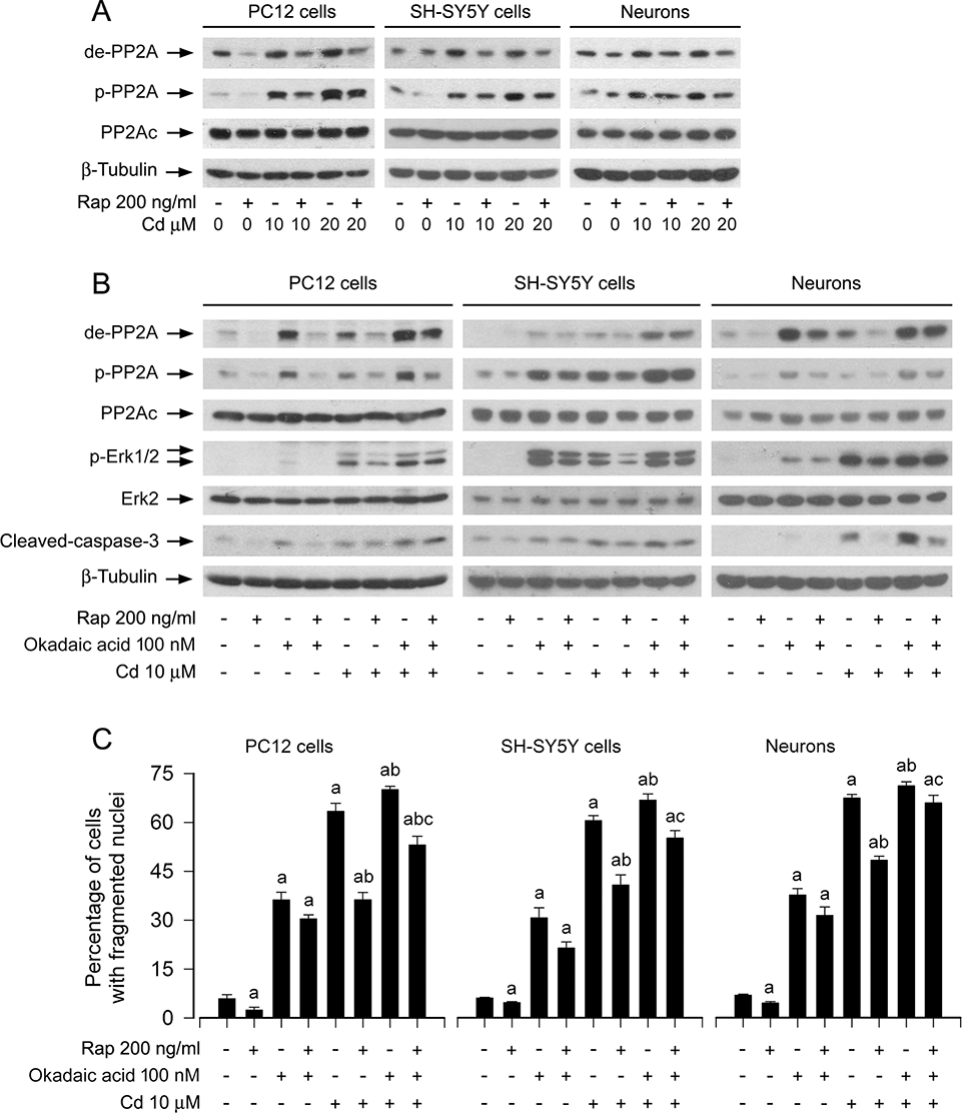
Rapamycin inhibits Cd-induced activation of Erk1/2 and apoptosis partially by preventing Cd from inactivating PP2A in neuronal cells PC12 cells, SH-SY5Y cells and primary neurons were pretreated with/without rapamycin (Rap, 200 ng/ml) for 48 h and then with/without okadaic acid (100 nM) for 1 h, followed by exposure to Cd (10 and/or 20 μM) for 4 h (for Western blotting) or 24 h (for cell apoptosis analysis). **A and B.** Total cell lysates were subjected to Western blotting using indicated antibodies, showing that rapamycin prevented Cd inactivation of PP2A (A). and pharmacological inhibition of PP2A with okadaic acid confered resistance to rapamycin's inhibition of Cd-induced PP2A inactivation, Erk1/2 phosphorylation in neuronal cells (B). **C.** Apoptotic cells were evaluated by nuclear fragmentation and condensation using DAPI staining. For (A) and (B), the blots were probed for β-tubulin as a loading control. Similar results were observed in at least three independent experiments. For (C), all data were expressed as means ± SE (*n* = 5). ^a^*P* < 0.05, difference with control group; ^b^*P* < 0.05, difference with 10 μM Cd group; ^c^*P* < 0.05, difference with Cd/Rap group or Cd/okadaic acid group.

It has been described that rapamycin can activate PP2A [39]. PP2A negatively regulates Erk1/2 pathway through dephosphorylation of Erk1/2 [35]. Our recent studies have demonstrated that Cd activates Erk1/2 pathway leading to apoptosis by inactivation of PP2A in neuronal cells [36], and the current study has found that rapamycin blocked Cd-induced activation of Erk1/2 pathway (Figures 2 and 3). Therefore, we postulated that rapamycin may inhibit Cd-activated Erk1/2 pathway by preventing Cd from inhibiting PP2A. To this end, PC12 cells, SH-SY5Y cells and primary neurons were pretreated with/without rapamycin (200 ng/ml) for 48 h, and then exposed to Cd (10 and 20 μM) for 4 h, following by Western blot analysis. As shown in Figure 4A, Cd and/or rapamycin did not apparently alter cellular protein levels of PP2Ac. However, pretreatment with rapamcyin obviously suppressed Cd-increased expression of demethylated-PP2A and phospho-PP2A (Figure 4A), two events that are related to decreased activity of PP2A [42], implying that rapamycin may inhibit Cd-induced activation of Erk1/2 and cell death partially by preventing Cd from inactivating PP2A in the neuronal cells.

To substantiate the role and significance of PP2A in rapamycin's inhibition of Cd-induced phospho-Erk1/2 and neuronal cell apoptosis, we used okadaic acid, a relatively specific PP2A inhibitor [43]. Because okadaic acid, at concentrations up to 100 nM, functions only as a selective inhibitor of PP2A without inhibiting PP1 in intact cells [43], PC2 cells, SH-SY5Y cells and primary neurons were pretreated with/without rapamycin (200 ng/ml) for 48 h, and then with/without okadaic acid (100 nM) for 1 h, followed by exposure to Cd (10 μM) for 4 h or 24 h. As shown in Figure 4B, Cd markedly induced the expression of demethylated-PP2Ac, phospho-PP2Ac and phospho-Erk1/2 in the absence or presence of okadaic acid. The effects appeared more potent in the cells co-treated with Cd/okadaic acid than in the ones treated with Cd or okadaic acid alone (Figure 4B). Okadaic acid alone elevated the basal levels of demethylated-PP2Ac, phospho-PP2Ac and phospho-Erk1/2 in the absence of rapamycin, and especially reversed the inhibitory effect of rapamycin on Cd-elicited events (Figure 4B). Furthermore, rapamycin inhibited the basal or Cd-induced cleaved-caspase-3 and cell apoptosis in PC12 cells, SH-SY5Y cells and primary neurons (Figure 4B and 4C). Okadaic acid alone significantly elevated the basal level of cell apoptosis and obviously strengthened Cd-induced apoptotic event (Figure 4B and 4C). Of note, okadaic acid conferred high resistance to rapamycin's inhibition of Cd-induced apoptosis in the cells (Figure 4B and 4C). These results support the notion that rapamycin inhibits Cd-induced Erk1/2 activation and apoptosis in part by activating PP2A in neuronal cells.

**Figure 5:**
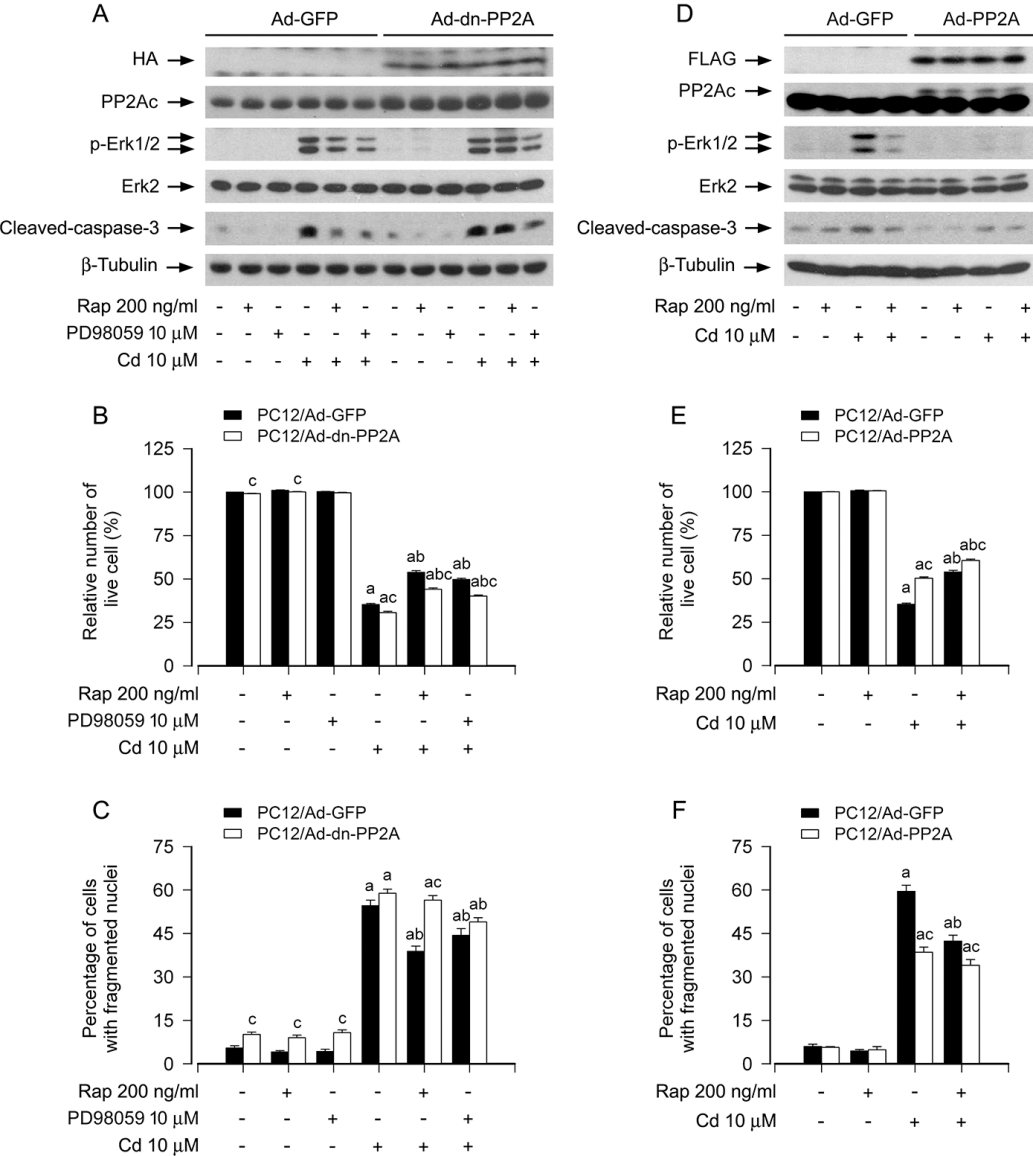
Ectopic expression of dominant negative PP2A or wild-type PP2A intervenes rapamycin blockage of Cd-induced Erk1/2 phosphorylation and apoptosis in neuronal cells PC12 cells, infected with Ad-dn-PP2A, Ad-PP2A and Ad-GFP (as control), respectively, were pretreated with/without rapamycin (Rap, 200 ng/ml) for 48 h or PD98059 (10 μM) for 1 h, followed by exposure to Cd (10 μM) for 4 h (for Western blotting) or 24 h (for live cell assay and cell apoptosis analysis). **A and D.** Total cell lysates were subjected to Western blotting using indicated antibodies. **B and E.** Live cells were detected by counting viable cells using trypan blue exclusion. **C and F.** Apoptotic cells were evaluated by nuclear fragmentation and condensation using DAPI staining. For (A) and (D), the blots were probed for β-tubulin as a loading control. Similar results were observed in at least three independent experiments. For (B), (C), (E), and (F), all data were expressed as means ± SE (*n* = 5). ^a^*P* < 0.05, difference with control group; ^b^*P* < 0.05, difference with 10 μM Cd group; ^c^*P* < 0.05, Ad-dn-PP2A group or Ad-PP2A group versus Ad-GFP group.

To confirm the above findings, PC12 cells, infected with Ad-dn-PP2A, Ad-PP2A and Ad-GFP (as control), respectively, were exposed to Cd (10 μM) for 4 h or 24 h following pretreatment with/without rapamycin (200 ng/ml) for 48 h or PD98059 (10 μM) for 1 h. As expected, a high level of HA-tagged dn-PP2A or FLAG-tagged PP2A-wt was seen in Ad-dn-PP2A- or Ad-PP2A-infected cells, but not in Ad-GFP-infected cells (control) (Figure 5A and 5D). Cd exposure was able to trigger phosphorylation of Erk1/2 in PC12/Ad-GFP and PC12/Ad-dn-PP2A cells (Figure 5A). Ectopic expression of dn-PP2A attenuated the inhibitory effect of rapamycin on Cd-induced phospho-Erk1/2, cleaved-caspase-3 and cell death (Figure 5A–5C). However, the cells expressing dn-PP2A remained sensitive to an Erk1/2 inhibitor, PD98059 (Figure 5A–5C). In contrast, over-expression of wild-type PP2A powerfully inhibited Cd-induced phospho-Erk1/2, cleaved-caspase-3 and cell apoptosis in the presence or absence of rapamycin, as detected by Western blotting, as well as live cell assay and DAPI staining (Figure 5D–5F). Taken together, our data strongly support that rapamycin blocks Cd-induced Erk1/2 activation and cell apoptosis in part via activation of PP2A in neuronal cells.

### Rapamycin prevents Cd-induced Erk1/2 activation and apoptosis also in part by blocking Cd down-regulation of PTEN and activation of Akt in neuronal cells

**Figure 6:**
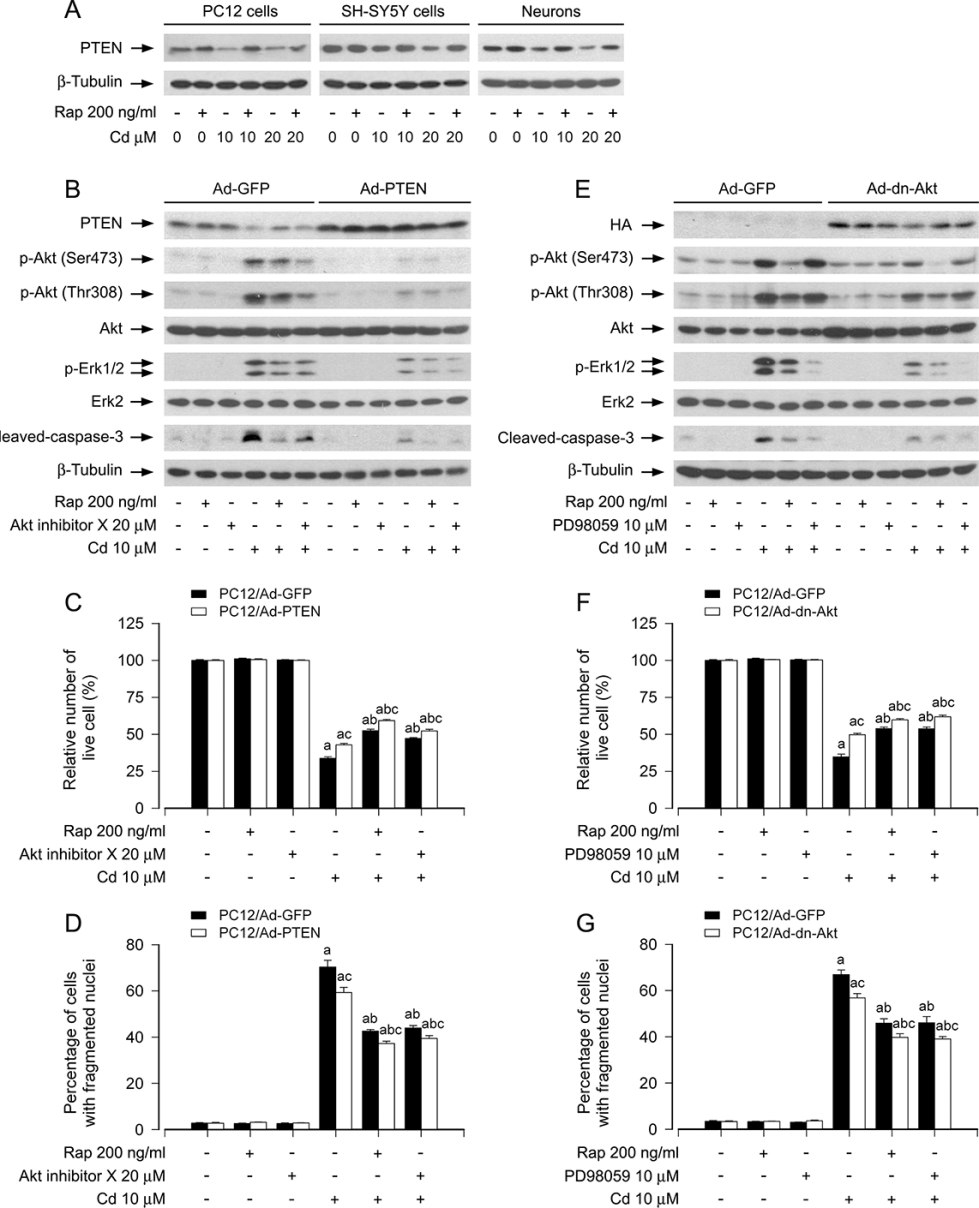
Rapamycin prevents Cd-induced Erk1/2 activation and apoptosis in part by blocking Cd down-regulation of PTEN and activation of Akt in neuronal cells PC12 cells, SH-SY5Y cells and primary neurons, or PC12 cells infected with Ad-PTEN, Ad-dn-Akt and Ad-GFP (as control), respectively, were pretreated with/without rapamycin (Rap, 200 ng/ml) for 48 h, or with/without Akt inhibitor X (20 μM) or PD98059 (10 μM) for 1 h, followed by exposure to Cd (10 and/or 20 μM) for 4 h (for Western blotting) or 24 h (for live cell assay and cell apoptosis analysis). **A, B and E.** Total cell lysates were subjected to Western blotting using indicated antibodies, showing that rapamycin prevented Cd down-regulation of PTEN in PC12 cells, SH-SY5Y cells and primary neurons (A). Over-expression of wild-type PTEN or dominant negative Akt, or inhibition of Akt with Akt inhibitor X strengthened rapamycin's inhibition of Cd-induced phospho-Erk1/2 in PC12 cells (B and E). **C and F.** Live cells were detected by counting viable cells using trypan blue exclusion. **D and G.** Apoptotic cells were evaluated by nuclear fragmentation and condensation using DAPI staining. For (A), (B), and (E), the blots were probed for β-tubulin as a loading control. Similar results were observed in at least three independent experiments. For (C), (D), (F), and (G), all data were expressed as means ± SE (*n* = 5). ^a^*P* < 0.05, difference with control group; ^b^*P* < 0.05, difference with 10 μM Cd group; ^c^*P* < 0.05, Ad-PTEN group or Ad-dn-Akt group versus Ad-GFP group.

It is well-known that PTEN negatively regulates Akt-mTOR pathway [22, 29, 37]. Emerging studies have suggested that PTEN may also negatively regulate Erk1/2 pathway in several malignancies [38]. In addition, PI3K/Akt may activate Erk1/2 through PKC [38]. Our recent studies have found that Cd down-regulation of PTEN results in activation of Akt/mTOR signaling and apoptosis of neuronal cells, which is attenuated by rapamycin [20]. Here, we hypothesized that rapamycin might block Cd-induced Erk1/2 activation and apoptosis also by modulating PTEN-Akt signaling in neuronal cells. In line with our previous finding [20], pretreatment with rapamycin (200 ng/ml) for 48 h potently prevented Cd-induced decrease of PTEN expression in PC12 cells, SH-SY5Y cells and primary neurons (Figure 6A). PC12 cells, infected with recombinant adenoviruses expressing wild-type human PTEN (Ad-PTEN) or Ad-GFP (as control), were pretreated with/without rapamycin (200 ng/ml) for 48 h or Akt inhibitor X (20 μM) for 1 h, followed by exposure to Cd (10 μM) for 4 h or 24 h. We observed that the infection with Ad-PTEN increased the expression of PTEN and slightly inhibited the basal levels of phosphorylation of Akt (Ser473 and Thr308), compared to the infection with Ad-GFP (Figure 6B). As expected, treatment with Cd decreased PTEN expression, and correspondingly increased phosphorylation of Akt and Erk1/2 in the control cells infected with Ad-GFP (Lane 4 vs. Lane 1). Over-expression of PTEN blocked Cd-induced phosphorylation of Akt and Erk1/2 (Lane 10 vs. Lane 4) (Figure 6B). Rapamycin, but not Akt inhibitor X, attenuated Cd-induced decrease in PTEN expression; both rapamycin and Akt inhibitor X diminished Cd-induced increase in Akt/Erk1/2 phosphorylation (Lane 5 vs. Lane 4, Lane 6 vs. Lane 4). Furthermore, over-expression of PTEN was able to potentiate the inhibitory effects of rapamycin or Akt inhibitor X on Cd-induced phosphorylation of Akt and Erk1/2 (Lane 11 vs. Lane 5, Lane 12 vs. Lane 6). Moreover, over-expression of PTEN also enhanced the protective effect of rapamycin or Akt inhibitor X against Cd-induced cleavage of caspase-3 (Lane 11 vs. Lane 5, Lane 12 vs. Lane 6) (Figure 6B). By live cell assay and DAPI staining, we observed that over-expression of PTEN alone partially prevented Cd-induced cell apoptosis in PC12 cells (Figure 6C and 6D). Addition of rapamycin or Akt inhibitor X elicited more significant protection against Cd-induced cell apoptosis (Figure 6C and 6D). Collectively, the findings support the notion that rapamycin inhibits Cd-induced activation of Erk1/2 and consequential cell apoptosis in neuronal cells, by preventing Cd down-regulation of PTEN and activation of Akt.

To further verify the role of Akt in rapamycin's blockage of Cd-induced Erk1/2 activation and apoptosis in neuronal cells, recombinant adenovirus expressing HA-tagged dominant negative Akt (Ad-dn-Akt) was utilized. As shown in Figure 6E, a high level of HA-tagged Akt mutant was seen in PC12 cells infected with Ad-dn-Akt, but not in the cells infected with Ad-GFP (control virus). Over-expression of dn-Akt remarkably suppressed Cd-triggered phosphorylation of Akt and Erk1/2 (Figure 6E). Rapamycin, but not PD98059, powerfully attenuated Cd-increased Akt phosphorylation. However, both rapamycin and PD98059 obviously inhibited Cd-induced Erk1/2 phosphorylation (Figure 6E). Interestingly, over-expression of dn-Akt was able to strengthen the inhibitory effects of rapamycin or PD98059 on Cd-induced phosphorylation of Akt or Erk1/2 (Figure 6E). Consistently, over-expression of dn-Akt also potently reinforced the preventive effects of rapamycin or PD98059 against Cd-induced cleavage of caspase-3 (Figure 6E), as well as cell apoptosis in PC12 cells (Figure 6F and 6G). These data clearly indicate that rapamycin blocks Cd-induced Erk1/2 activation and apoptosis by preventing Cd activation of Akt in neuronal cells.

### Inhibition of mTOR kinase activity is necessary for rapamycin's activation of PP2A, up-regulation of PTEN and inactivation of Akt, leading to suppression of Erk1/2 and cell apoptosis in Cd-exposed neuronal cells

**Figure 7:**
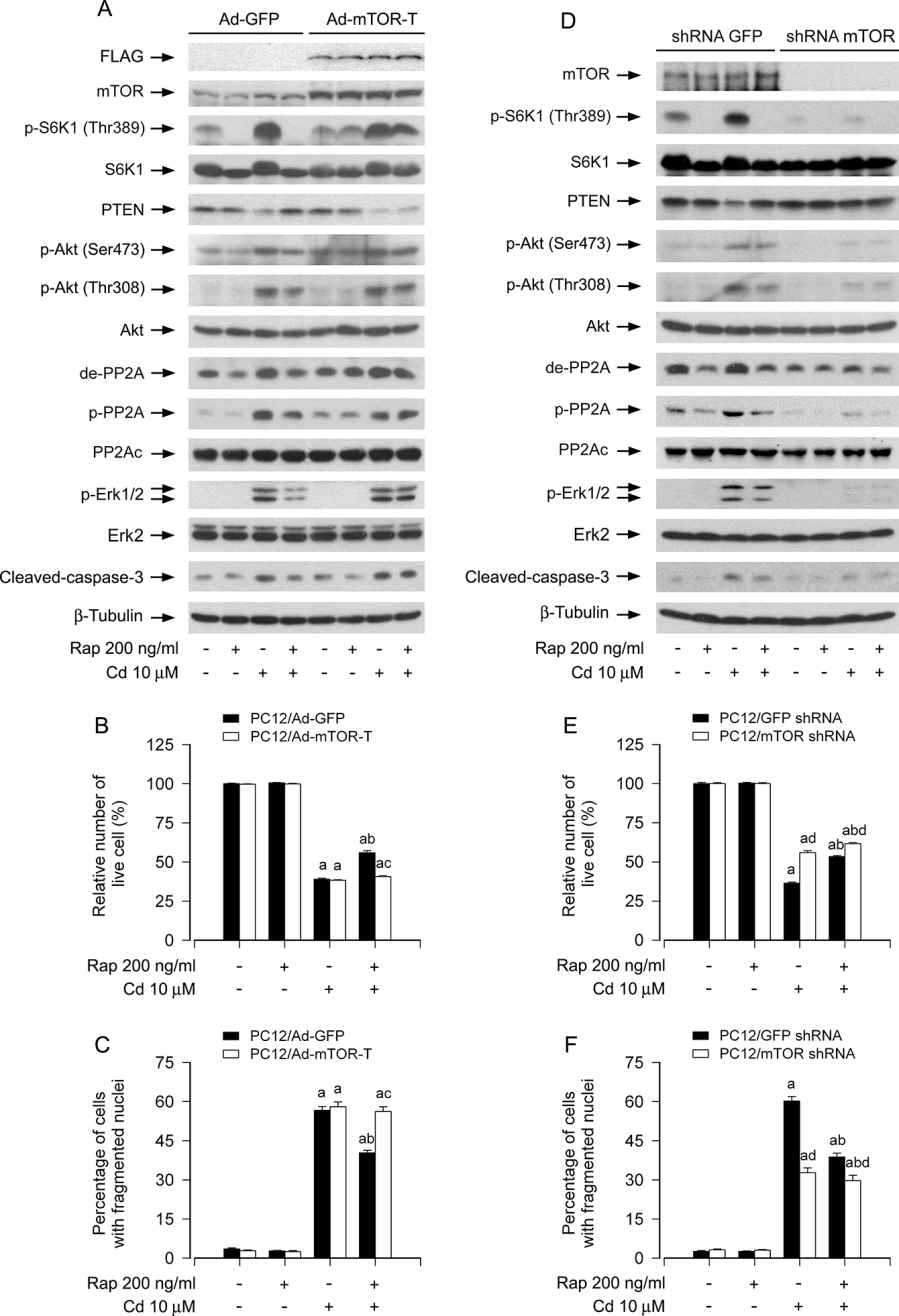
Rapamycin mediates activation of PP2A, up-regulation of PTEN and inactivation of Akt, leading to inhibition of Cd-induced Erk1/2 phosphorylation and apoptosis in neuronal cells in an mTOR kinase activity-dependent manner PC12 cells infected with Ad-mTOR-T or Ad-GFP (as control), or PC12 cells infected with lentiviral shRNA to mTOR or GFP, respectively, were pretreated with/without rapamycin (Rap, 200 ng/ml) for 48 h, followed by exposure to Cd (10 μM) for 4 h (for Western blotting) or 24 h (for live cell assay and cell apoptosis analysis). **A and D.** Total cell lysates were subjected to Western blotting using indicated antibodies. **B and E.** Live cells were detected by counting viable cells using trypan blue exclusion. **C and F.** Apoptotic cells were evaluated by nuclear fragmentation and condensation using DAPI staining. For (A) and (D), the blots were probed for β-tubulin as a loading control. Similar results were observed in at least three independent experiments. For (B), (C), (E), and (F), all data were expressed as means ± SE (*n* = 5). ^a^*P* < 0.05, difference with control group; ^b^*P* < 0.05, difference with 10 μM Cd group; ^c^*P* < 0.05, Ad-mTOR-T group versus Ad-GFP group; ^d^*P* < 0.05, mTOR shRNA group versus GFP shRNA group.

To uncover whether rapamycin prevents Cd-induced inactivation of PP2A, down-regulation of PTEN, and activation of Akt, leading to suppression of Cd-induced Erk1/2 activation and neuronal apoptosis, is through inhibition of mTOR activity, PC12 cells were infected with recombinant adenoviruses expressing GFP (Ad-GFP, as control) or FLAG-tagged rapamycin-resistant and kinase-active mTOR (S2035T, Ad-mTOR-T) for 24 h, and then pretreated with/without rapamycin (200 ng/ml) for 48 h, followed by exposure to Cd (10 μM) for 4 h or 24 h. The function of Ad-mTOR-T was confirmed by determining the expression of FLAG-tagged mTOR mutant and the phosphorylation level of S6K1. We observed that ectopic expression of FLAG-tagged mTOR-T, but not GFP, conferred high resistance to rapamycin inhibition of phosphorylation of S6K1 in PC12 cells (Figure 7A), as seen in other cell lines [44, 45]. Of interest, expression of FLAG-mTOR-T, but not GFP, rendered substantial resistance to rapamycin's inhibitory effects on Cd-induced demethylated-PP2Ac, phospho-PP2Ac, down-regulated PTEN, phospho-Akt, phospho-Erk1/2 and cleaved-caspase-3, leading to reduced apoptosis in Cd-exposed PC12 cells (Figure 7A–7C), implying that rapamycin activates PP2A, upregulates PTEN and inactivates Akt, thereby inhibiting Cd-induced activation of Erk1/2, as well as neuronal apoptosis, in an mTOR kinase activity-dependent manner.

We also confirmed the above findings using RNA interference. As demonstrated in Figure 7D, lentiviral shRNA to mTOR, but not GFP, silenced expression of mTOR protein by ~90% in PC12 cells, as detected by Western blotting. Down-regulation of mTOR significantly decreased the mTOR kinase activity, since the basal or Cd-induced phosphorylation of S6K1 (Thr389), routinely used as an indicator of mTOR kinase activity [16, 17], was almost not detectable by Western blotting (Figure 7D). Of note, down-regulation of mTOR blocked Cd-induced demethylated-PP2Ac, phospho-PP2Ac, down-regulated PTEN, phospho-Akt, and phospho-Erk1/2 in the cells even without pretreatment with rapamycin (Figure 7D). Furthermore, as expected, down-regulation of mTOR obviously prevented Cd-induced cleavage of caspase-3 and cell apoptosis, and potentiated the inhibitory effect of rapamycin (Figure 7D–7F). Taken together, our data underscore the concept that mTOR is a hub of the PP2A/PTEN/Akt/Erk network involved in Cd-induced neuronal cell death.

## DISCUSSION

Cadmium, as one of the most toxic environmental and industrial pollutants, is mainly released from smelting and refining of metals, burning of chemical fuels and municipal wastes, and cigarette smoking [1, 12]. Cd can penetrate the blood-brain barrier and accumulate into the brain contributing to the development of CNS damages [7, 46]. Cd toxicity causes brain cellular dysfunction, lethal cerebral edema and parkinsonism [9, 15]. Studies have shown that cerebral cortical and hippocampal neurons are targets of Cd toxicity, which is thought to play an important role in human neurodegenerative diseases [7–9, 12–15]. Therefore, it is of great importance to find effective treatments against the damage of Cd on brain neurons in individuals with Cd-induced neurodegenerative diseases. Rapamycin is not only a lipophilic macrolide antibiotic but also a specific mTOR inhibitor [30]. A series of studies have recently shown that rapamycin is useful in treatment of several human diseases, such as cancer, diabetes, obesity, genetic disorders, and neurological diseases [47, 48]. Especially, rapamycin has been reported to be an effective treatment in several experimental models of neurodegenerative diseases, including PD, AD and HD [26, 48–50], implying that rapamycin may act as a pharmacological compound with therapeutic benefits for fighting neurodegenerative diseases.

Our recent studies have shown that exposure of Cd to mice or neuronal cells (PC12 cells, SH-SY5Y cells or primary neurons) results in brain damage and neuronal cell apoptosis in part through activation of Akt/mTOR signaling pathway [12, 28]. By inhibiting activation of Akt/mTOR pathway, rapamycin *in vitro* and *in vivo* effectively attenuates Cd-triggered neuronal cell death [12, 28]. We have also identified that Cd activates Erk1/2 pathway contributing to neuronal cell death [28, 36]. However, little is known about whether and how rapamycin rescues cells from Cd-induced cell death by inhibiting Erk1/2 pathway. Here we provide evidence that rapamycin prevented Cd-induced apoptotic cell death by inhibiting Erk1/2 pathway in neuronal cells. Further, we found that rapamycin inhibited Cd activation of Erk1/2 via preventing Cd-inactivation of PP2A and PTEN signaling network.

In this study, we found that rapamycin inhibits Cd-induced phosphorylation of Erk1/2 and cleavage of caspase-3 in PC12 cells, SH-SY5Y cells and primary neurons, as detected by Western blotting. This is further supported by the results of phospho-Erk1/2 immunofluorescence staining and caspase3/7 activity assay. To corroborate the above findings, pharmacological Erk1/2 inhibitor PD98059 was utilized. We noticed that the combination of rapamycin with PD98059 exhibited a more potent inhibitory effect on Cd-induced activation of Erk1/2 and caspase-3, as well as cell apoptosis than rapamycin or PD98059 alone (Figure 3B and 3C). Further, silencing Erk1/2 remarkably blocked Cd-induced phosphorylation of Erk1/2 and cleavage of caspase-3 (Figure 3D). Concurrently, silencing Erk1/2 conferred substantial resistance to Cd-induced neuronal apoptosis, as evidenced by the reduced percentages of cells with nuclear fragmentation and condensation in PC12 cells. Of importance, addition of rapamycin reinforced the events, in line with the data obtained from the co-treatment with rapamycin/PD98059. Collectively, our findings indicate that rapamycin prevents Cd-induced apoptosis, at least in part, by blocking activation of Erk1/2 pathway in neuronal cells.

Rapamycin has been documented to activate PP2A [39]. It is known that PP2A negatively regulates Erk1/2 pathway through dephosphorylation of Erk1/2 [35]. We have demonstrated that Cd activates Erk1/2, in part via inhibition of PP2A, in neuronal cells [36]. This led us to investigate the effect of rapamycin on PP2A activity in neuronal apoptosis triggered by Cd. In this study, we did not observe that rapamycin altered cellular protein expression of the catalytic subunit (PP2Ac) (Figure 4A). However, we found that Cd induced robust expression of demethylated-PP2Ac and phospho-PP2Ac (Tyr307), which was attenuated by rapamycin in PC12 cells, SH-SY5Y cells and primary neurons (Figure 4A). These data indicate that rapamycin activates the phosphatase activity of PP2A at least by inhibiting Cd-elevated demethylation and phosphorylation of PP2Ac, two events responsible for PP2A inactivation [42]. Next, we tested the hypothesis that rapamycin prevents Cd-induced Erk1/2 activation and apoptosis via PP2A-dependent mechanism in neuronal cells. For this, pharmacological/genetic inhibition or rescue experiments for PP2A were carried out, respectively. We found that over-expression of wild-type PP2Ac potentiated rapamycin's suppression of Cd-induced phosphorylation of Erk1/2 and neuronal apoptosis, whereas inhibition of PP2A by okadaic acid, or expression of dominant negative (dn)-PP2A resulted in the robust phosphorylation of Erk1/2 and conferred high resistance to rapamycin inhibition of Cd-induced cell apoptosis. Furthermore, we also noticed that the cells expressing dn-PP2A were able to remain sensitive to MEK1/2 inhibitor PD98059. In contrast, over-expression of wild-type PP2A markedly inhibited Cd-induced phosphorylation of Erk1/2 and cell apoptosis in the presence or absence of rapamycin. These results support a model in which rapamycin blocks Cd-induced neuronal apoptosis by preventing Cd activation of Erk1/2, which is partially attributed to inactivation of PP2A.

PTEN is well-known to negatively regulate Akt/mTOR pathway [22, 29, 37]. Our recent studies have noticed that Cd down-regulates PTEN protein expression, leading to activation of Akt/mTOR signaling in PC12 cells [20]. In this study, pretreatment with rapamycin (200 ng/ml) for 48 h potently prevented Cd from reducing PTEN expression in PC12 cells, SH-SY5Y cells and primary neurons (Figure 6A). Of note, emerging studies have suggested that PTEN also negatively regulates Erk1/2 pathway in several malignancies [38]. Additionally, PI3K/Akt can activate Erk1/2 through PKC [38]. Putting all data together, we postulated that a cross-talk may occur between PTEN, Akt and Erk1/2 pathways in neuronal cells in response to Cd, i.e. Cd down-regulation of PTEN and concurrent activation of Akt may result in activation of Erk1/2, which may be prevented by rapamycin. In this study, for the first time, we presented evidence that rapamycin inhibited Cd-induced neuronal apoptosis indeed by preventing Cd down-regulation of PTEN and activation of Akt, resulting in inhibition of Erk1/2 pathway. This is strongly supported by the findings that ectopic expression of wild-type PTEN or dominant negative Akt, or inhibition of Akt with Akt inhibitor X enhanced rapamycin's inhibitory effect on Cd-induced phospho-Erk1/2 and cell death in PC12 cells (Figure 6B–6G). Our data underscore that rapamycin has an ability to prevent Cd from inactivation of PTEN and activation of Akt, thereby attenuating Cd-induced Erk1/2 activation and neuronal apoptosis.

Rapamycin is a well-known mTOR inhibitor [16, 17, 30]. However, studies have also shown that rapamycin suppresses differentiation of C2C12 cells [45], which is mTOR kinase activity-independent, although this remains controversial [45, 51]. This prompted us to study whether rapamycin prevents Cd inhibition of PP2A, downregulation of PTEN and activation of Akt, resulting in activation of Erk1/2 and cell apoptosis in an mTOR kinase activity-dependent manner. We found that rapamycin failed to block Cd inactivation of PP2A, downregulation of PTEN, and activation of Akt, and thus could not prevent Cd-induced Erk1/2 activation and cell apoptosis in Ad-mTOR-T-infected cells, but not in Ad-GFP-infected control cells, suggesting an mTOR-dependent mechanism involved. This is further supported by the findings that silencing mTOR resulted in activation of PP2A, restoration of PTEN expression, and inhibition of Akt, thereby inhibiting Erk1/2 activation and cell apoptosis. These results clearly indicate that inhibition of mTOR, following rapamycin treatment, can indirectly suppress Erk1/2 pathway, attenuating Cd-induced neuronal apoptosis. It is worth mentioning that MEK/Erk1/2 can also indirectly activate mTOR pathway in a cell type-specific manner [52]. For instance, oncogenic MEK/Erk1/2 signaling stimulates mTORC1 activity by promoting p90 ribosomal S6 kinase (RSK)-mediated raptor phosphorylation [53]. Phorbol ester (PMA), a tumor promoter, hyper-activates the MEK/Erk1/2 pathway and increases phosphorylation of S6K/S6 in SKBR3 breast cancer cells, which can be abrogated by co-treatment with U0126 and rapamycin [54]. Taken together, there exists a complex cross-talk between MEK/Erk1/2 and mTOR pathways. Further research may unveil more mechanisms by which mTOR and Erk1/2 regulate each other.

How does rapamycin suppress Cd-induced activation of mTOR involved in PP2A inactivation and PTEN reduction? It has been reported that the activity of PTEN could be almost completely abolished by hydrogen peroxide (H_2_O_2_), a well-known oxidant, *in vitro* and *in vivo* [55]. Additionally, the activity of PP2A is inhibited by H_2_O_2_ in SK-N-SH neuroblastoma cells [56] and in PC12 cells and primary neurons [57] as well. The data suggest that oxidative stress inactivates cellular PTEN and PP2A. In line with the above findings, Cd-induced reactive oxygen species (ROS) also down-regulates the protein expression of PTEN and the activity of PP2A in PC12 cells, SH-SY5Y cells and primary neurons [20, 36]. Of note, growing evidence has shown that rapamycin has the ability to attenuate oxidative stress-induced cell damage or death [12, 20, 58, 59]. For example, rapamycin inhibits H_2_O_2_-induced loss of vascular contractility [59]. Diabetes-induced oxidative retinal injury is ameliorated by rapamycin [58]. Recent studies from our group have revealed that pretreatment with rapamycin *in vitro* for 48 h prevents Cd-induced ROS from inactivation of PTEN and activation of Akt/mTOR pathway, as well as neuronal cell death [20]. Administration of rapamycin *in vivo* also potently attenuates Cd-induced ROS activation of Akt/mTOR contributing to brain damage and neuron death in mice [12]. It has been observed that rapamycin suppresses Cd-induced ROS, by down-regulating the expression of ROS generating enzyme NADPH oxidase 2 (NOX2) and its regulatory proteins (p22^phox^, p67^phox^, p40^phox^, p47^phox^, and Rac1) in PC12 and SH-SY5Y cells [20], suggesting that mTOR may positively regulate expression of these ROS generating proteins, although the mechanism remains to be defined. In the current study, we have demonstrated that rapamycin can inhibit mTOR-dependent PP2A/PTEN/Akt/Erk network contributing to Cd-induced neuronal cell apoptosis. Thus, we tentatively deduce that rapamycin is likely to act by the mechanism that counteracts Cd-induced oxidative stress by down-regulating expression of NOX2 and its regulatory proteins, thereby not only preventing Cd-induced activation of mTOR, but also blocking Cd-induced inactivation of PP2A and down-regulation of PTEN. Rapamycin may be involved in modulating interactions of diverse signals and expression of genes associated with Cd-induced neurotoxicity. Undoubtedly, more studies are needed to address these issues.

**Figure 8:**
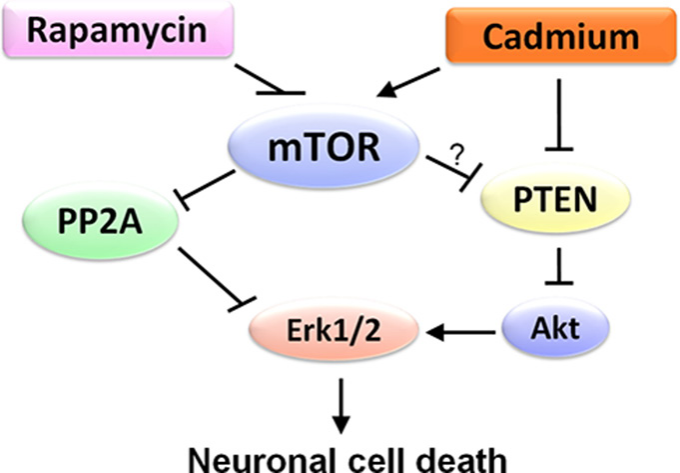
A schematic diagram showing how rapamycin inhibits Erk1/2-mediated neuronal apoptosis caused by Cd Rapamycin blocks Cd-induced Erk1/2 activation, thereby attenuating Cd-induced apoptosis. Mechanistically, rapamycin inhibits Cd activation of Erk1/2, not only by preventing Cd inhibition of PP2A, but also through hindering Cd down-regulation of PTEN and activation of Akt in an mTOR kinase activity-dependent manner in neuronal cells.

In summary, here we have identified that rapamycin attenuates Cd-induced neuronal apoptosis by blocking Erk1/2 pathway. Rapamycin blocks Cd-induced Erk1/2 activation contributing to apoptosis not only by preventing Cd inhibition of PP2A, but also through hindering Cd down-regulation of PTEN and activation of Akt in neuronal cells in an mTOR kinase activity-dependent manner (Figure 8). The results indicate that rapamycin inhibits Cd activation of Erk1/2-mediated neuronal apoptosis, at least in part, through targeting mTOR-PP2A/PTEN signaling network. Our findings suggest that rapamycin has a potential application in prevention of Cd-induced neurodegenerative disorders.

## MATERIALS AND METHODS

### Reagents

Cadmium chloride, 4′, 6-diamidino-2-phenylindole (DAPI), poly-D-lysine (PDL), Okadaic acid, PD98059, and protease inhibitor cocktail were purchased from Sigma (St Louis, MO, USA). Rapamycin was from ALEXIS (San Diego, CA, USA). Dulbecco's modified Eagle medium (DMEM), 0.05% Trypsin-EDTA, NEUROBASAL™ Media, and B27 Supplement were purchased from Invitrogen (Grand Island, NY, USA). Horse serum and fetal bovine serum (FBS) were supplied by Hyclone (Logan, UT, USA). Enhanced chemiluminescence solution was from Millipore (Billerica, MA, USA). Akt inhibitor X was provided by Santa Cruz Biotechnology (Santa Cruz, CA, USA). The following antibodies were used: PP2ACα (BD Biosciences, San Jose, CA, USA); phospho-S6K1 (Thr389), phospho-Akt (Ser473), and cleaved-caspase-3 (Cell Signaling Technology, Beverly, MA, USA); demethylated-PP2A, Akt, phospho-Erk1/2 (Thr202/Tyr204), Erk2, and S6K1 (Santa Cruz Biotechnology, Santa Cruz, CA, USA); phospho-PP2A, and PTEN (Epitomics, Burlingame, CA, USA); phospho-Akt (Thr308), FLAG, HA, mTOR, and β-tubulin (all from Sigma); goat anti-rabbit IgG-horseradish peroxidase (HRP), goat anti-mouse IgG-HRP, and rabbit anti-goat IgG-HRP (Pierce, Rockford, IL, USA). Other chemicals were purchased from local commercial sources and were of analytical grade.

### Cell lines, primary neurons and cultures

Rat pheochromocytoma (PC12) and human neuroblastoma SH-SY5Y cell lines were from American Type Culture Collection (ATCC) (Manassas, VA, USA), which were seeded in a 6-well or 96-well plate pre-coated with (for PC12) or without (for SH-SY5Y) PDL (0.2 μg/ml). PC12 cells were cultured in antibiotic-free DMEM supplemented with 10% horse serum and 5% FBS, whereas SH-SY5Y cells were grown in antibiotic-free DMEM supplemented with 10% FBS. Cells were maintained in a humid incubator (37°C, 5% CO_2_).

To isolate primary neurons, female ICR mice were purchased from from the Laboratory Animal Center, Nanjing Medical University (Nanjing, China). Animals were handled in accordance with the guidelines of the Institutional Animal Care and Use Committee, and were in compliance with the guidelines set forth by the Guide for the Care and Use of Laboratory Animals. Primary cortical neurons were isolated from fetal mice at 16–18 days of gestation as described [60]. Afterwards, cells were seeded in a 6-well or 96-well plate pre-coated with 10 μg/ml PDL and cultured in NEUROBASAL™ Media (Invitrogen) supplemented with 2% B27 Supplement (Invitrogen), 2 mM glutamine (Invitrogen), 1 mM sodium pyruvate (Invitrogen), 5 μg/ml insulin (Sigma), and 40 μg/ml of gentamicin (Invitrogen), and grown in a humid incubator (37°C, 5% CO_2_). Fresh medium was replaced every 3 days. The primary neurons were used for experiments after 6 days of culture.

### Recombinant adenoviral constructs and infection of cells

The recombinant adenoviruses expressing wild-type human PTEN (Ad-PTEN), FLAG-tagged rapamycin-resistant and kinase-active mTOR (S2035T, designated Ad-mTOR-T), FLAG-tagged wild-type rat PP2ACα (Ad-PP2A), hemagglutinin (HA)-tagged dominant-negative (dn) PP2A catalytic subunit (PP2Ac) (L199P) (dn-PP2A), and the control vector expressing green fluorescent protein (GFP) alone (Ad-GFP) were described previously [20, 44, 57, 61]. Recombinant adenovirus encoding HA-tagged dominant negative Akt (dn-Akt, T308A/S473A) was a generous gift from Dr. Kenneth Walsh (Boston University, Boston, MA). For experiments, PC12 cells were grown in the growth medium and infected with the individual adenovirus for 24 h at 5 of multiplicity of infection (MOI = 5). Subsequently, cells were used for experiments. Ad-GFP served as a control. Expression of HA-tagged dn-PP2A and dn-Akt, as well as FLAG-tagged PP2A and mTOR-T was determined by Western blot analysis with antibodies to HA and FLAG, respectively. As S6K1 is a substrate of mTOR, the function of mTOR-T in the cells was assessed by immunoblotting with antibodies to phospho-S6K1 (Thr389).

### Lentiviral shRNA cloning, production, and infection

Lentiviral shRNAs to mTOR, Erk1/2 and GFP (for control) were generated and used as described [28].

### Live cell assay by trypan blue exclusion

Parental PC12 cells, PC12 cells infected with lentiviral shRNA to mTOR or GFP, or PC12 cells infected with Ad-PTEN, Ad-dn-Akt, Ad-mTOR-T, Ad-dn-PP2A, Ad-PP2A or Ad-GFP, respectively, were seeded at a density of 5 × 10^5^ cells/well in a PDL-coated 6-well plate. Next day, cells were treated with/without Cd (10 and/or 20 μM) for 24 h following pre-incubation with/without rapamycin (200 ng/ml) for 48 h, or with/without Akt inhibitor X (20 μM) or PD98059 (10 μM) for 1 h, with 5 replicates of each treatment. Subsequently, live cells were monitored by counting viable cells using trypan blue exclusion test.

### Assays for cell morphology and caspase-3/7 activity

PC12 cells, SH-SY5Y cells and primary neurons were seeded in a PDL-uncoated or -coated 6-well plate (5 × 10^5^ cells/well) or 96-well plate (1 × 10^4^ cells/well). The next day, the cells were exposed to Cd (10 and/or 20 μM) for 24 h following pre-incubation with/without rapamycin (200 ng/ml) for 48 h, with 5 replicates of each treatment. Subsequently, the images for morphological analysis were taken under an Olympus inverted phase-contrast microscope (Olympus Optical Co., Melville, NY, USA) (200 ×) equipped with the Quick Imaging system. Caspase-3/7 activity was determined using Caspase-Glo^®^ 3/7 Assay Kit (Promega, Madison, WI, USA), following the instructions of the supplier.

### Immunofluorescence staining

PC12 cells, SH-SY5Y cells and primary neurons were seeded at a density of 5 × 10^5^ cells/well in a 6-well plate containing a PDL-uncoated or -coated glass coverslip per well. Next day, after exposed to Cd (10 and 20 μM) for 24 h following pre-incubation with/without rapamycin (200 ng/ml) for 48 h, cells were fixed with 4% paraformaldehyde and incubated with 3% normal goat serum, followed by adding mouse anti-phospho-Erk1/2 antibody (1:50, diluted in PBS containing 1% BSA) for overnight at 40°C, and then incubating with secondary antibody to FITC-conjugated goat anti-mouse IgG (1:500, diluted in PBS containing 1% BSA) for 1 h at room temperature, as described [62]. Finally, slides were mounted in glycerol/PBS (1:1, v/v) containing 2.5% 1, 4-diazabiclo-(2, 2, 2)octane. Cell images were captured under a fluorescence microscope (Nikon 80i, Tokyo, Japan) equipped with a digital camera. For quantitative analysis of the fluorescence staining, the integral optical density (IOD) was measured by Image-Pro Plus 6.0 software (Media Cybernetics Inc., Newburyport, MA, USA).

### DAPI staining

PC12 cells, SH-SY5Y cells and primary neurons, or PC12 cells infected with lentiviral shRNA to mTOR, Erk1/2 or GFP, or PC12 cells infected with Ad-PTEN, Ad-dn-Akt, Ad-mTOR-T, Ad-dn-PP2A, Ad-PP2A or Ad-GFP, respectively, were seeded at a density of 5 × 10^5^ cells/well in a PDL-uncoated or -coated 6-well plate. The next day, cells were treated with/without Cd (10 and/or 20 μM) for 24 h following pre-incubation with/without rapamycin (200 ng/ml) for 48 h, or with/without Akt inhibitor X (20 μM) or PD98059 (10 μM) for 1 h, with 5 replicates of each treatment. In some cases, cells were pretreated with/without rapamycin (200 ng/ml) for 48 h, and then with/without okadaic acid (100 nM) or PD98059 (10 μM) for 1 h, followed by exposure to Cd (10 μM) for 24 h. Afterwards, the cells with fragmented and condensed nuclei were determined using DAPI staining as described [36]. Photographs were taken with a fluorescence microscope (Nikon 80i, Japan) equipped with a digital camera.

### TUNEL staining

PC12 cells, SH-SY5Y cells and primary neurons, seeded at a density of 5 × 10^5^ cells/well in a 6-well plate containing a PDL-coated glass coverslip per well, were treated with/without Cd (10 and 20 μM) for 24 h post pre-incubation with/without rapamycin (200 ng/ml) for 48 h, followed by the terminal deoxynucleotidyl transferase (TdT)-mediated deoxyuridine triphosphate (dUTP) nick-end labeling (TUNEL) staining, according to the manufacture's instructions of *In Situ* Cell Death Detection Kit^®^ (Roche, Mannheim, Germany). Finally, all stained samples were analyzed by fluorescence microscopy (Nikon 80i, Japan) equipped with digital camera. For quantitative analysis of the fluorescence intensity, IOD was measured by Image-Pro Plus 6.0 software as described above.

### Western blot analysis

After treatments, the indicated cells were briefly washed with cold PBS, and then on ice, lysed in the radioimmunoprecipitation assay buffer. Afterwards, Western blotting was performed as described previously [36].

### Statistical analysis

Results were expressed as mean values ± standard error (Means ± SE). Student's *t*-test for non-paired replicates was used to identify statistically significant differences between treatment means. Group variability and interaction were compared using either one-way or two-way ANOVA followed by Bonferroni's post-tests to compare replicate means. Significance was accepted at *P* < 0.05.
